# The use of time‐of‐flight camera to assess respiratory rates and thoracoabdominal depths in patients with chronic respiratory disease

**DOI:** 10.1111/crj.13581

**Published:** 2023-01-29

**Authors:** Olivier Van Hove, Vasileios Andrianopoulos, Ali Dabach, Olivier Debeir, Alain Van Muylem, Dimitri Leduc, Alexandre Legrand, Rudy Ercek, Véronique Feipel, Bruno Bonnechère

**Affiliations:** ^1^ Department of Pneumology Erasme Hospital Brussels Belgium; ^2^ Institute for Pulmonary Rehabilitation Research Schoen Klinik Berchtesgadener Land Schoenau am Koenigssee Germany; ^3^ LISA ‐ Laboratory of Image Synthesis and Analysis Université Libre de Bruxelles Brussels Belgium; ^4^ Laboratory of Cardiorespiratory Physiology Université Libre de Bruxelles Brussels Belgium; ^5^ Department of Respiratory Physiology, Pathophysiology and Rehabilitation Research Institute for Health Sciences and Technology, University of Mons Mons Belgium; ^6^ Laboratory of Functional Anatomy Université Libre de Bruxelles Brussels Belgium; ^7^ REVAL Rehabilitation Research Center, Faculty of Rehabilitation Sciences Hasselt University Diepenbeek Belgium; ^8^ Technology‐Supported and Data‐Driven Rehabilitation, Data Sciences Institute Hasselt University Diepenbeek Belgium

**Keywords:** assessment, breathing, Kinect sensor, respiratory diseases, validation

## Abstract

**Introduction:**

Over the last 5 years, the analysis of respiratory patterns presents a growing usage in clinical and research purposes, but there is still currently a lack of easy‐to‐use and affordable devices to perform such kind of evaluation.

**Objectives:**

The aim of this study is to validate a new specifically developed method, based on Kinect sensor, to assess respiratory patterns against spirometry under various conditions.

**Methods:**

One hundred and one participants took parts in one of the three validations studies. Twenty‐five chronic respiratory disease patients (14 with chronic obstructive pulmonary disease (COPD) [65 ± 10 years old, FEV_1_ = 37 (15% predicted value), VC = 62 (20% predicted value)], and 11 with lung fibrosis (LF) [64 ± 14 years old, FEV_1_ = 55 (19% predicted value), VC = 62 (20% predicted value)]) and 76 healthy controls (HC) were recruited. The correlations between the signal of the Kinect (depth and respiratory rate) and the spirometer (tidal volume and respiratory rate) were computed in part 1. We then included 66 HC to test the ability of the system to detect modifications of respiratory patterns induced by various conditions known to modify respiratory pattern (cognitive load, inspiratory load and combination) in parts 2 and 3.

**Results:**

There is a strong correlation between the depth recorded by the Kinect and the tidal volume recorded by the spirometer: *r* = 0.973 for COPD patients, *r* = 0.989 for LF patients and *r* = 0.984 for HC. The Kinect is able to detect changes in breathing patterns induced by different respiratory disturbance conditions, gender and oral task.

**Conclusions:**

Measurements performed with the Kinect sensors are highly correlated with the spirometer in HC and patients with COPD and LF. Kinect is also able to assess respiratory patterns under various loads and disturbances. This method is affordable, easy to use, fully automated and could be used in the current clinical context.

Respiratory patterns are important to assess in daily clinics. However, there is currently no affordable and easy‐to‐use tool to evaluate these parameters in clinics. We validated a new system to assess respiratory patterns using the Kinect sensor in patients with chronic respiratory diseases.

## Graphical abstract

The non‐contact respiratory pattern measurement system presented in this paper is easy to use, non‐invasive, and doesn't need to be calibrated, and can therefore be easily used in the daily clinic. This type of technique allows the measurement of changes in the thoracoabdominal depth induced by different types of breathing and/of pathologies. This system should be used in the future to assess the patients quickly and on a regular basis. It allows the detection of variations in respiratory patterns and its asynchrony over time and in relation to the constraints on the respiratory system.

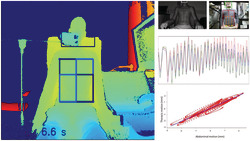

## 1 INTRODUCTION

The assessment and evaluation of breathing patterns are becoming more and more popular in both research and clinical environments. The air volume that is transported into and out of the lungs during a cycle of breathing (tidal volume) in non‐intubated subjects presents many opportunities in evaluating the effectiveness of a treatment or assessing the severity of several common respiratory pathologies, such as asthma, chronic obstructive pulmonary disease (COPD) and pulmonary fibrosis (PF). Spirometer is still considered as the gold standard to perform assessment and evaluation of breathing patterns (e.g. tidal volume and respiratory rate). However, there are some limitations with the use of this kind of device. First, it requires a mouthpiece, a filter and a nose clip, and it has been previously shown that such a methodology may significantly affect the respiratory patterns.^1^ In order to overcome these limitations, other methods have been developed, such as plethysmography cabins,^2^ inductive plethysmography,^3^ and optoelectronic plethysmography.^4^ Although these techniques do not require a mouthpiece, they have some other limitations (e.g. no transportability, installation time and high cost).

Currently, an important area of research is the development of non‐contact methods (i.e., marker less motion assessment) for lung function analysis. Such methods allow an accurate analysis of respiratory patterns by limiting instrumental effects (i.e., no mouthpiece or nose clip)^1^ and can accurately estimate slow or forced vital capacity (VC, but not forced expiratory volume, in 1 s (FEV1).^5^ Furthermore, the evaluation can be performed without touching the patients, which is of particular importance in the context of the COVID‐19 pandemic.

Non‐contact methods also allow to visualise thoracoabdominal asynchrony,^6^ which is affected by changing positions and pathologies. For example, in more than half of COPD patients, there is a strong asynchrony in the supine position, whereas in a seated position, the rib cage and abdomen are synchronous.^7^ Another important advantage is that those systems can be used in rehabilitation to assess the thoracoabdominal coordination efforts of COPD patients^8^ or the effects of ventilation.^9^ However, because of their high price, poor transportability and the need of health‐care professional to perform these assessments, these analysis are limited to a few specialised centres, and few patients can benefit from this type of examination.

Since the release of the first version of the Kinect™ sensor in 2010, researchers and clinicians have directly felt the possible potential of this device in clinics.^10^ There are, however, only a few studies on the validation of the Kinect to evaluate respiratory patterns or breathing volume, and most of those works have been done for different radiotherapy‐based applications, such as respiratory tracking and collision detection,^11^, ^12^ not to assess pulmonary functions.

Currently, there is a lack of information about the use of the Kinect sensor to assess respiratory patterns in healthy subjects and patients suffering from chronic respiratory diseases under various conditions. One study previously demonstrated that the Kinect was sensitive enough to detect different externally induced airway obstructions.^13^ Therefore, the objective of this study was to validate the use of the Kinect camera as a non‐invasive respiratory motion‐tracking system. To do so, we first compare the results of the Kinect with a spirometer in patients suffering from chronic diseases (part 1). Then we evaluate the system's ability to capture changes in the respiratory patterns induced by several perturbations such as cognitive and/or cognitive loads (CLs) in healthy individuals (parts 2 and 3).

## 2 MATERIALS AND METHODS

### 2.1 Participants

For part 1, 25 patients with chronic respiratory diseases (14 COPD and 11 patients with PF) and 10 healthy controls were recruited from an outpatient clinic (Cliniques Universitaires Bruxelles, Erasme University Hospital, Brussels, Belgium). For parts 2 and 3, healthy subjects were included. The characteristics of the healthy participants and patients are presented in Table 1. This study was approved by the Ethical Committee of Erasme Hospital (B406201734629, B406201733566 and B406201838283 for parts 1–3, respectively), and written informed consent was obtained from all subjects prior to their participation.

**TABLE 1:** The mean (SD) characteristics of the subjects and patients included in the different protocols

Parameters	Part 1			Part 2	Part 3
	COPD	PF	Healthy subjects	Healthy subjects	Healthy subjects
***N***	14	11	10	34	32
**Male**	10	7	5	17	22
**Age, years**	65 (10)	64 (14)	25 (3)	24 (2)	26 (4)
**Height, cm**	166 (10)	170 (12)	169 (10)	172 (10)	174 (11)
**Weight, kg**	74 (32)	76 (18)	73 (12)	68 (14)	68 (4)
**BMI, kg/m2**	24 (6)	26 (6)	25 (5)	23 (3)	22 (3)
**FEV**_**1**_ **, %pred**	37 (15)	55 (19)	NA	NA	NA
**VC, %pred**	62 (20)	62 (20)	NA	NA	NA

*Notes*: Data are presented as the mean value (SD). VC (%pred), vital capacity in percentage of the predicted value; FEV_1_(%pred), forced expiratory volume during the first second in percentage of the predicted value. COPD: chronic obstructive pulmonary disease, PF: pulmonary fibrosis.

### 2.2 Experimental setup

Kinect V2 (Microsoft, Redmond, WA, USA) was used. This camera measures the distance between the sensor and the surface of objects in the sensor's field of view using time‐of‐flight technology for every pixel within the depth frame at a rate of 30 fps. The depth camera has a resolution of 512 × 424 and has the ability to detect distances ranging from 0.5 to 4.5 m.^14^ This method uses amplitude‐modulated waves and has a millimetric resolution.^15^ There are several advantages of this method. For example, no calibration is needed^16^; it also offers marker‐free acquisition and greater precision of measurements.

The cameras were located at a height of 1 m and at a distance of 1.5 m from the subject, and this distance seems, indeed, to be optimal for depth estimation.^17^ The position of the subject during the recording was chosen according to the position described by Niérat et al, ‘*sit upright in a high‐backed chair with their neck in a neutral position and their back as straight as possible. They were also asked not to move*.’^1^

**FIGURE 1:**
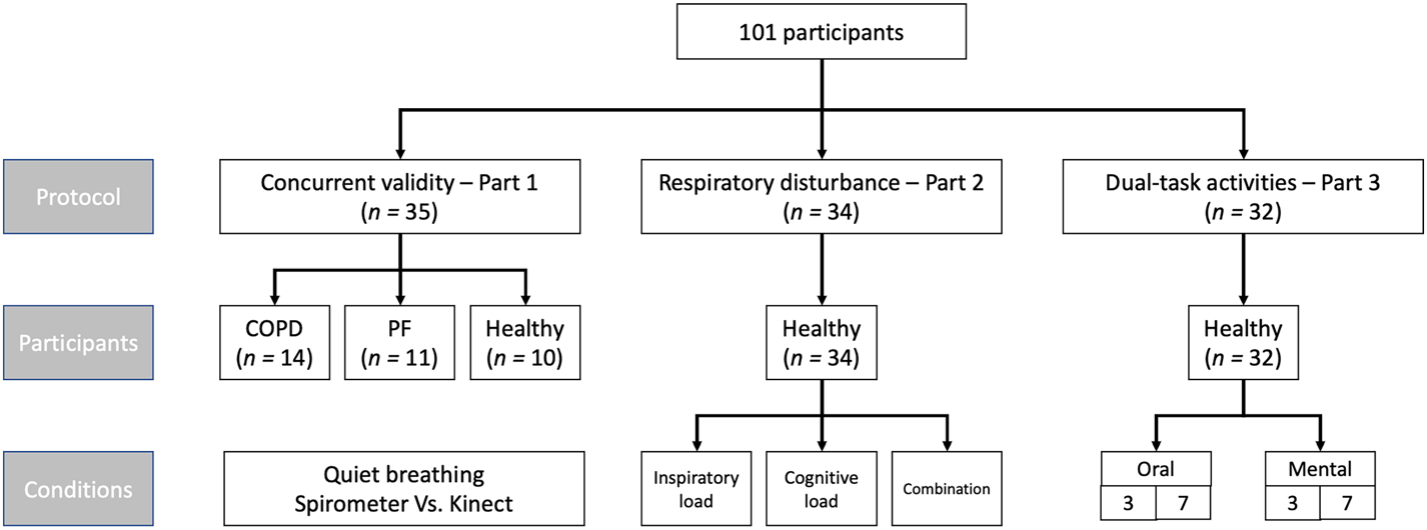
Flow chart of the participants included in the different parts of this study

We performed a triple step validations study to analyse to what extent this solution could be used. Flow of study design and the participants in the different part of the studies are presented in Figure 1.

### 2.3 Software implementation

The implementation is based on a previously validated method.^18^

**FIGURE 2:**
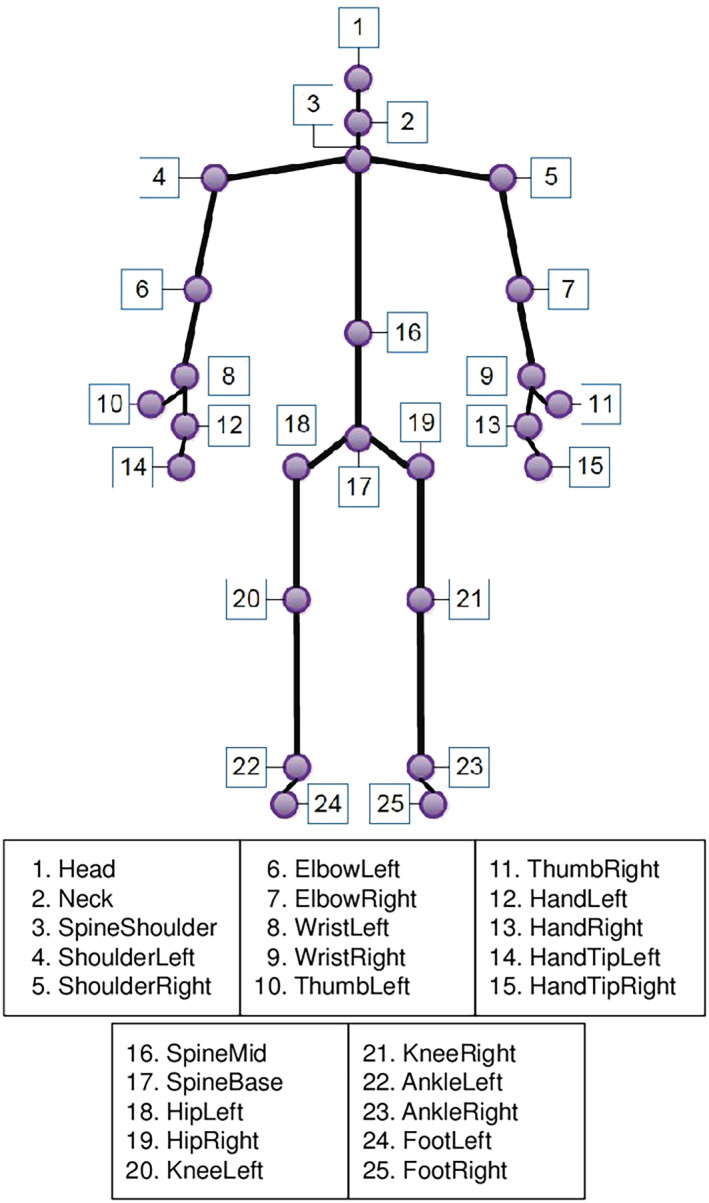
Skeletal landmark definition provided by the Kinect software development kit

**FIGURE 3:**
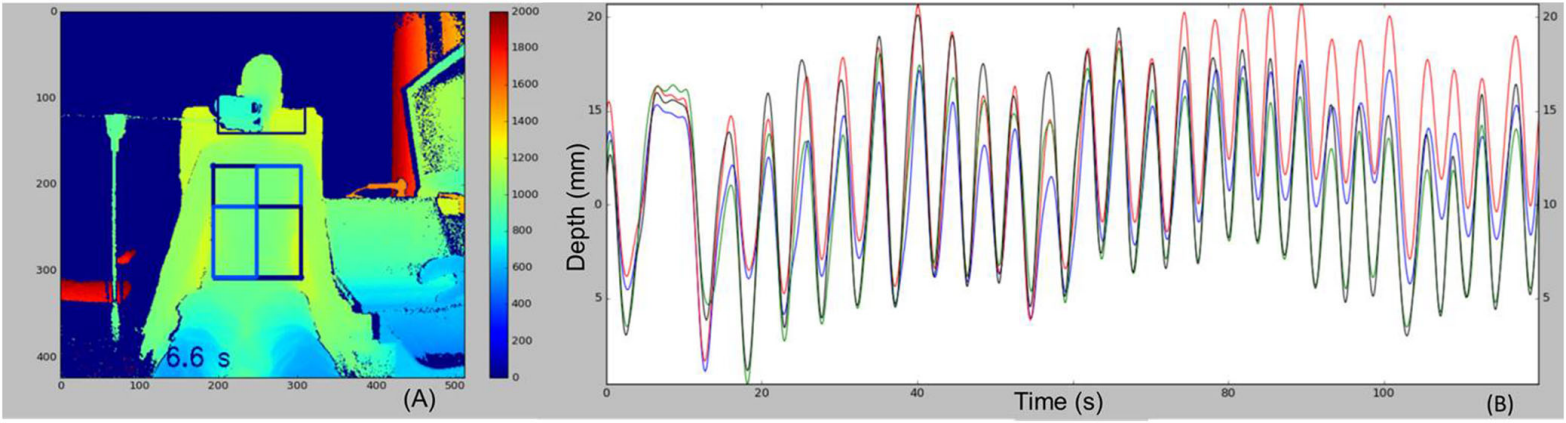
(A) Depth image with illustration of the regions of interests (ROIs). In orange, the ROIs are defined by default; in red, the ROIs are defined by the proposed method. (B) Examples of the curves obtained using different ROIs

Briefly, the points of the region of interest (ROI) are SpineMid and shoulderRight, ShoulderLeft, HipRight or HipLeft (Figure 2). In this case, the rectangles defining the abdominal ROI are much smaller than those defining the thoracic ROI. This can strongly limit the region of abdominal analysis. To correct this, the same width (along the *x* axis) was used to define the rectangles by considering the *x* coordinate of the ShoulderRight/ShoulderLeft point instead of the *x* coordinate of the HipRight/HipLeft point. The dividing line between the thorax and the abdomen is defined by a horizontal line passing through the point SpineMid. (Figure 3A,B). The ROIs are defined inside the thorax and abdomen. An estimation of the volumes has been developed considering the surface of the ROI multiplied by the variation of depth.

### 2.4 Kinect indices

Based on the image analysis described above, three variables are computed to evaluate the respiratory patterns.

**FIGURE 4:**
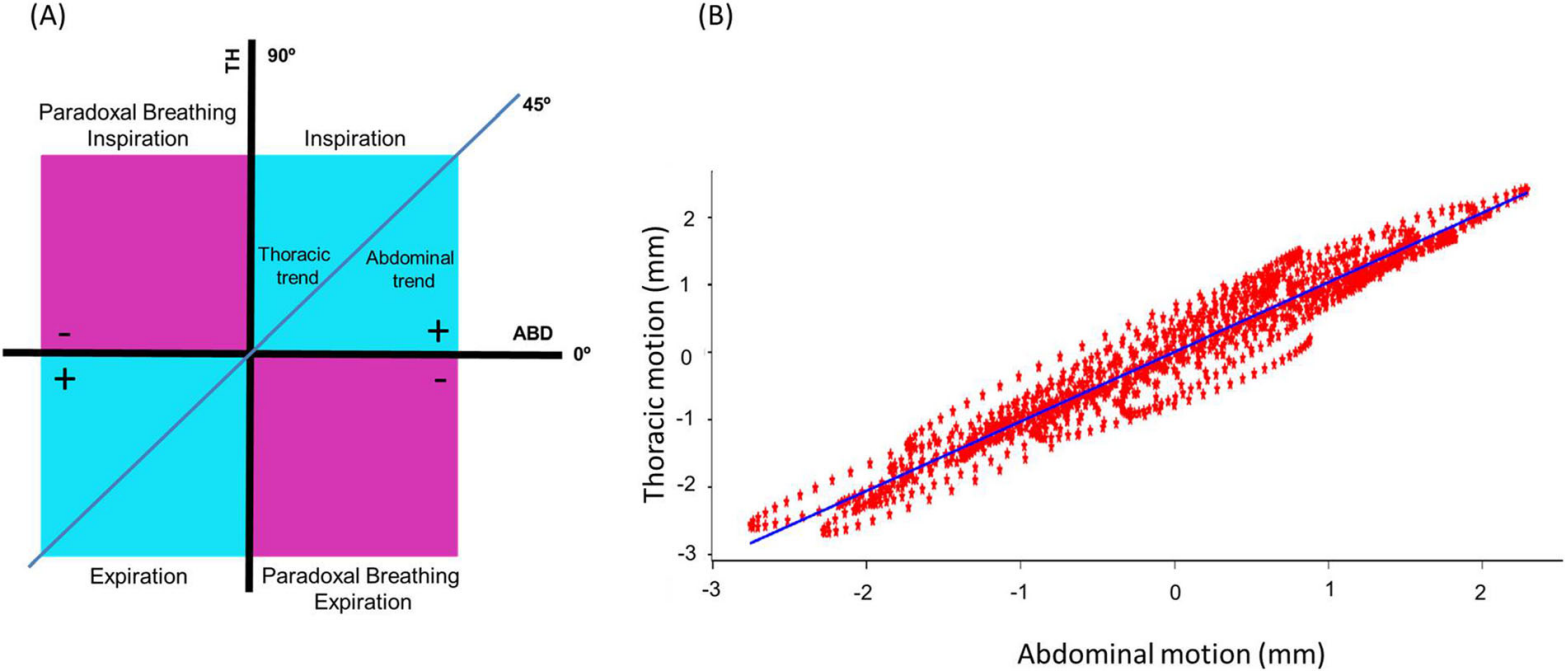
(A) Interpreting the interpolation of the thoracic and abdominal motion. ABD, abdominal motion (Depth abdominal); TH, thoracic motion (Depth thoracic); +, positive angle of the straight line; −, negative angle of the straight line. (B) Example of regression thoracic and abdominal motions (depth)

1. *Depth*: The mean depths of the ROI (variations in ROI depth over time; see Figure 2B)
2. Respiratory rates
3. *The thoracoabdominal trend*: to estimate thoracoabdominal contributions, a regression analysis was performed between thoracic and abdominal motions during a given recording (Figure 4A), and the angle between the regression line and the *x* axis was computed. An angle of 45° (first bisector) represents similar displacements of the thorax and the abdomen. An angle greater or smaller than 45° represents a thoracic or an abdominal preponderance for a given recording

### 2.5 Validations process

To validate the newly developed solutions, we performed a three‐step validation (Figure 1). First, we performed a concurrent validation study of this system compared to the spirometry in patients with chronic respiratory diseases (part 1). Because it is difficult to assess patients when they are experiencing exacerbation phases,^19^ we artificially modified the respiratory patterns of healthy participants by inducing different perturbations in parts 2 and 3.

#### 2.5.1 Part 1: Concurrent validity

The patients were asked to breath as normally and comfortably as possible, for 3 min, through a mouthpiece (wearing a nose clip to avoid potential compensation) in a handheld spirometer (hand‐held spirometer/USB Pocket‐Spiro® MPM100 MEC Medical Electronic Construction R&D). Kinect and spirometer measurements were recorded simultaneously. To assess the impact of clothes on the quality of the depth estimation, measurements were performed with and without t‐shirt. For the spirometer, the data extracted from the breathing pattern are tidal volume and respiratory rate. This data will be compared to the depth and respiratory rate recorded by the Kinect.

In order to compare both signals, several steps are needed. First, the signals are ‘*detrended’* (by subtracting the best linear trend from the data). This allows one to extract the variation of the signals and easily compare them. This step corrects the drift signal of the spirometer. Then, a frequency filter (low pass from 0 to 1 Hz) is applied to the signals. A 1 Hz value was chosen considering the respiratory rate interval of a healthy adult, which varies from 0.2 to 0.34 Hz (FR of 12 to 20 cycles per minute).^18^

This filtration process helps to mitigate related disturbances of the Kinect measurement noise. However, the sampling of the Kinect signal is not constant (the sampling frequency varies from 20 to 30 Hz). Therefore, it is necessary to homogenise the sampling before applying filtration. This is carried out with a sampling frequency of 20 Hz. The data of the spirometer are also sampled with the same frequency (instead of 100 Hz). This makes it easy to determine the time lag between the spirometer and the Kinect. The cross‐correlation process is then applied to both signals to find the time offset. When the signals are synchronised, peak detection (extrema) is applied (only synchronous peaks are considered). Subsequently, a linear regression is performed between the peaks of the signal from the Kinect and those from the spirometer.

#### 2.5.2 Part 2: Respiratory disturbance

In this part, we evaluate the Kinect's ability to capture known changes in respiratory patterns under different types of loads. Three situations were tested: CL, inspiratory load (IL) and both combined. These situations were then compared to the baseline situation (rest).

For the CL, we used a cognitive mobile game.^20^ In this game, subjects must correctly classify items on the right or left side of the screen. This game is inspired by the go/no go test and challenge response control task shifting.

For the IL, the subjects had to breathe throughout a mouthpiece and throughout an inspiratory threshold load (MAS Philips Respironics Threshold IMT Lung Muscle Trainer Adjustable Constant Pressure) at 15 cmH_2_O. The subjects were equipped with a nose clip to avoid nasal compensation.

Each recording time lasted 3 min, and the order of the tasks was randomly defined to minimise the risk of bias.

In order to estimate the instrumental effect, the subjects were asked, after each recording, to evaluate the awareness of breath on a 10‐cm Visual Analogue Scale. We use the same protocol as Garfinkel et al. for the perception of heartbeat (complete confidence/a full perception of heartbeat),^21^ but we modified it for breathing (0 = complete confidence, 10 = a full perception of breathing).

#### 2.5.3 Part 3: Dual‐task activities

Finally, we evaluated the Kinect's ability to detect changes in respiratory patterns and thoracoabdominal coordination when subjects are asked to perform cognitive tasks. It has been indeed demonstrated than cognition interferes with breathing.^22^

In addition to the baseline condition, two cognitive tasks were asked of the subjects^23^:

1. *Mental cognitive task*: a mental calculation using the subtraction of 3 (mental 3) and a mental calculation using the subtraction of 7 (mental 7).
2. *Oral cognitive task*: an oral calculation using a subtraction of 3 (oral 3) and an oral calculation using a subtraction of 7 (oral 7).

### 2.6 Statistical analysis

The normality of each parameter was checked using graphical methods (box plots, histograms and QQ‐plots), as well as Kolmogorov–Smirnov tests.

For the first protocol, Pearson's correlation coefficient (*r*) was used to compare the results of the Kinect and the spirometer. We used the average peak‐to‐peak values of the different cycles to compare the two systems. Then we applied linear regression to assess the relationship in the two group of patients (COPD and PF) as well as the potential influence of the clothing (with and without t‐shirt) on this relationship.

For the second protocol, we used a non‐parametric method because the data were not normally distributed. Kruskal–Wallis tests were performed to test different conditions. A Dunn test was applied during post‐hoc analysis to determine the differences between the conditions.

For the third protocol, two‐way ANOVA tests were applied to compare the five conditions, as well as the gender and potential interactions between the conditions and gender. Bonferroni tests were used to correct for multiple comparisons in our post‐hoc analysis.

Statistical analyses were performed at an overall significance level of 0.05.

## 3 RESULTS

### 3.1 Part 1: Concurrent validity

**FIGURE 5:**
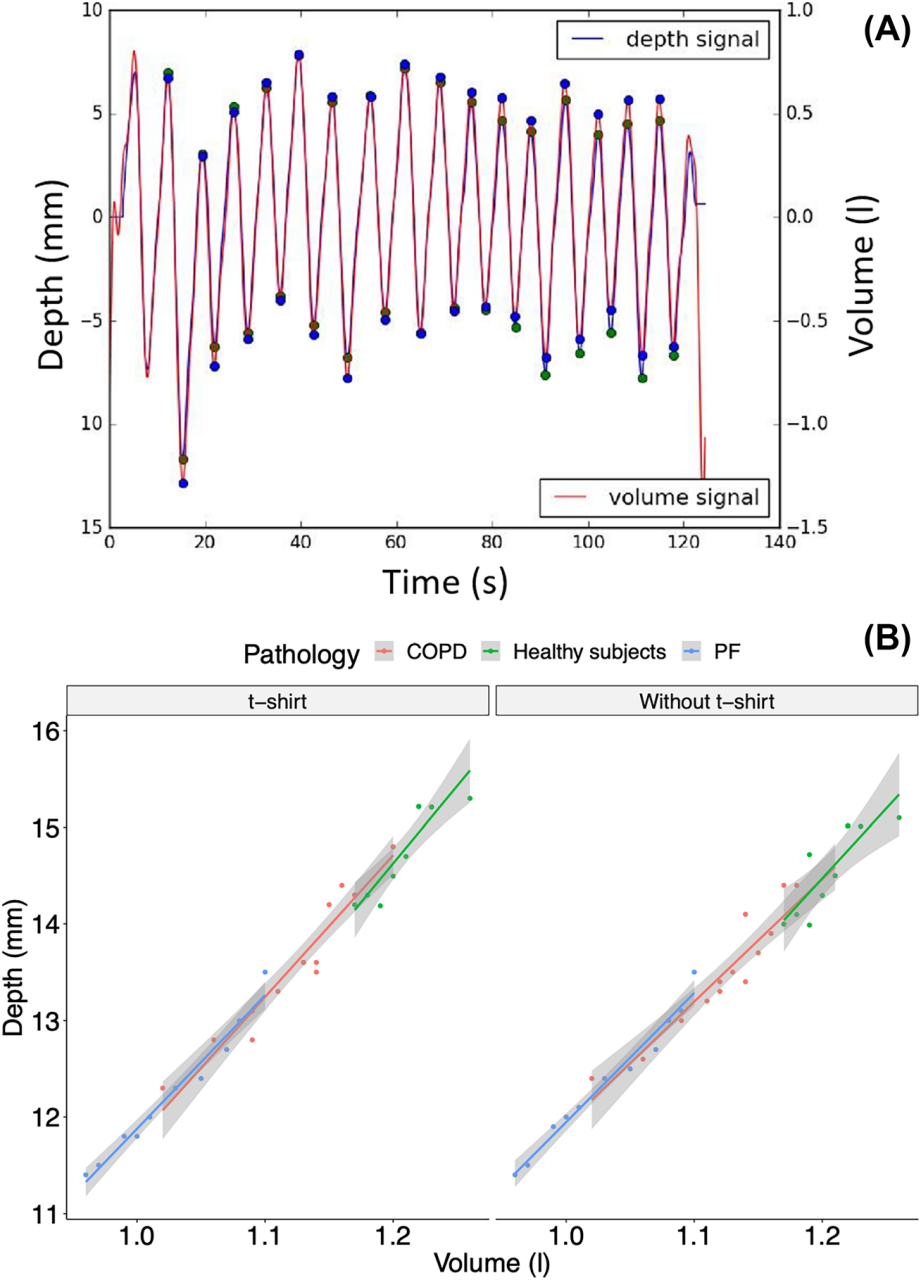
(A) Kinect and spirometer signals after the superposition and marking of inspiratory and expiratory peaks for each of the two signals. (B) Correlations between the spirometer and the Kinect sensors for the different populations with and without t‐shirt

There is a strong correlation between the volume recorded by the spirometer and the depth recorded by the Kinect for healthy subjects (*r* = 0.984, *p* < 0.001 and *r* = 0.973, *p* < 0.001, with and without a t‐shirt, respectively) as well as for COPD patients (*r* = 0.985, *p* < 0.001 and *r* = 0.989, *p* < 0.001, with and without a t‐shirt, respectively) and patients with PF (*r* = 0.988, *p* < 0.001 and *r* = 0.989, *p* < 0.001, with and without a t‐shirt, respectively), see Figure 5. We then computed linear regression for the three group and did not find difference between the pathologies with (*β* [95%CI] = 0.071 [0.069–0.074] for healthy subjects, 0.072 [0.069–0.075] for COPD patients and 0.072 [0.069–0.076] for PF patients) or without t‐shirt (*β* [95%CI] = 0.072 [0.069–0.075] for healthy subjects, 0.072 [0.070–0.076] for COPD patients and 0.073 [0.070–0.076] for PF patients).

### 3.2 Part 2: Respiratory disturbance

Complete results are presented in Table 2, which illustrates the significant effect of the conditions (*p* < 0.001). We observed a highly significant decrease in respiratory depth with the addition of a CL and a highly significant increase with the inspiratory and combined loads. There was a highly significant increase in the respiratory rate associated with the CL and a significant decrease with the inspiratory and combined loads. The Minute Ventilation Like (VM_like_) (mean depth multiplied by the respiratory rate and expressed as a unitless value) is also modified by the conditions. During CL, we observe a significant decrease in VM_like_ (*p* < 0.001), whereas for IL and combined we have a significant increase in VM_like_ (*p* < 0.001).

**TABLE 2:** The mean (SD) results for the cycle time, the respiratory rate, and the depth

Parameter	Rest	Cognitive load		Inspiratory load		Combination	
	Mean (SD)	Mean (SD)	*p*‐value	Mean (SD)	*p*‐value	Mean (SD)	*p*‐value
**Time of Cycle (s)**	3.9 (1.2)	3.0 (0.5)	<0.001	4.8 (1.8)	0.008	4.6 (1.7)	0.048
**Respiratory rate** **(cycles/min)**	15 (3.4)	19 (3.0)	<0.001	13 (4.1)	0.006	13 (3.9)	0.043
**Depth (mm)**	5.0 (2.5)	3.5 (1.9)	<0.001	9.0 (5)	< 0.001	8.1 (3.9)	0.001
**Minute Ventilation Like**	73.8 (21.4)	65.3 (11.8)	<0.001	116.4 (37.8)	<0.001	102.1 (23.3)	<0.001

*Note*: The *p*‐values are the results of the post hoc corrections compared with the rest value.

For the instrumental effect, the baseline value was 5.5 (2.6). The consciousness of the breathing was significantly lower under CL (1.5 [1.8], *p* < 0.001) and higher under IL (7.8 [2.2], *p* < 0.001) but did not differ under a combination of both (5.7 [2.4], *p* = 0.89).

### 3.3 Part 3: Dual‐task activities

The mean depths during the different tasks are presented in Figure 5. Only the oral 7 shows a highly significant increase in depth. The mental cognitive task shows a tendency to decrease depth but not significantly.

**FIGURE 6:**
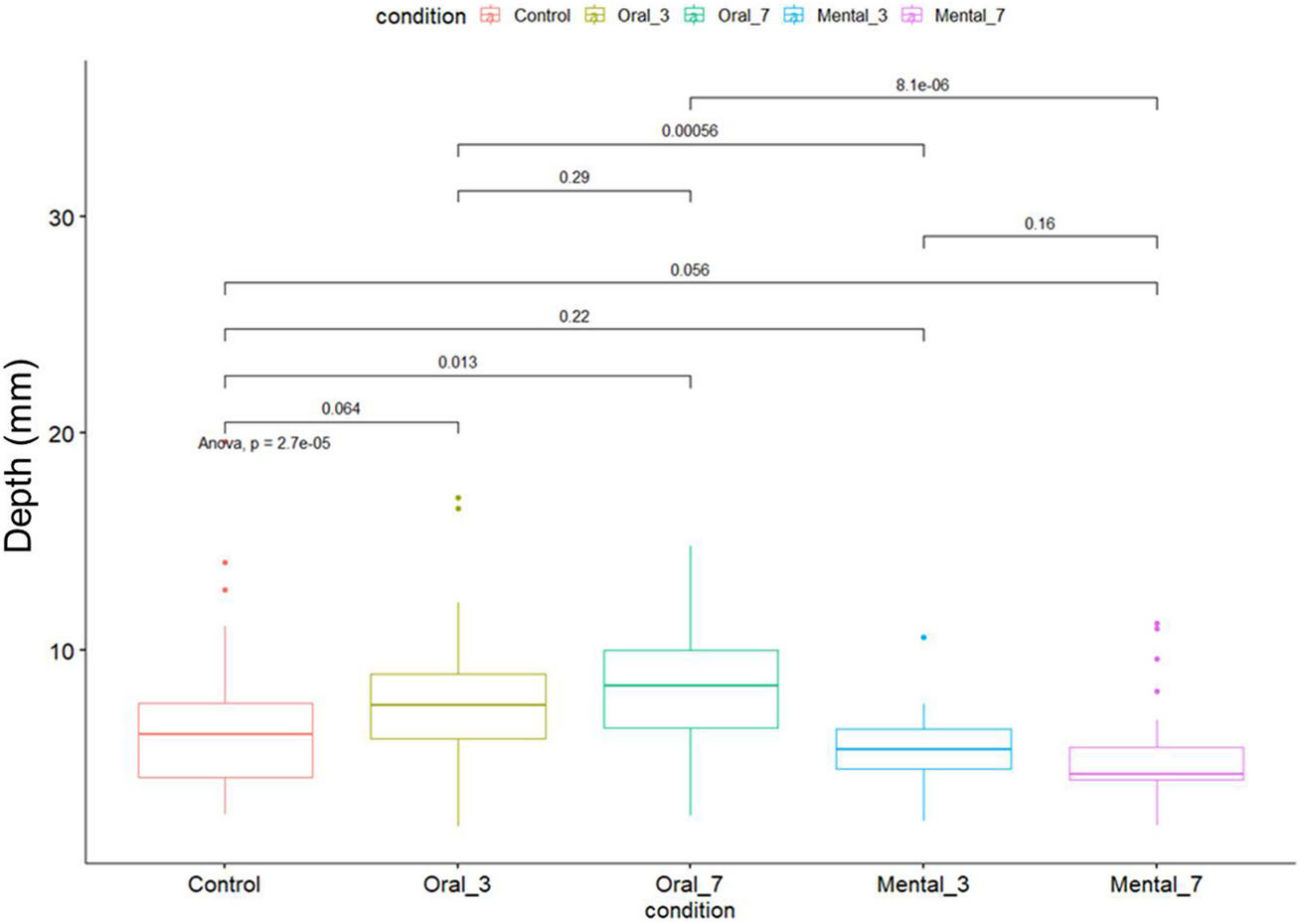
Boxplots of depth for the different conditions and results of Bonferroni's corrections

If you compare the cognitive conditions between them, there is a highly statistically significant difference between mental 3 and oral 3 and a very highly statistically significant difference between mental 7 and oral 7 (Figure 6).

The variations of the thoracoabdominal angles are presented in Table 3. There is no effect for the conditions (*p* = 0.52) but a highly significant effect for gender (*p* < 0.001), with women presenting higher values than men. The interaction between the conditions and gender is not significant (*p* = 0.51).

**TABLE 3:** The mean (SD) results for the trend angle between the thorax and the abdomen for the five different conditions for the whole group and then for male and female separately

Group	Rest	Mental 3	Mental 7	Oral 3	Oral 7
**Whole group (*n =* 32)**	47 (13)	50 (10)	48 (11)	52 (12)	51 (15)
**Female (*n =* 11)**	54 (9)	54 (10)	52 (11)	59 (7)	52 (20)
**Male (*n =* 21)**	45 (13)	49 (10)	46 (11)	49 (13)	51 (13)

*Note*: There is no effect for the conditions (*p* = 0.52) but a highly significant effect for gender (*p* < 0.001).

## 4 DISCUSSION

The aim of this study was to validate the use of the Kinect sensor to assess respiratory patterns under various conditions, either for different clinical conditions or in disturbing situations known to modify respiratory patterns. The main finding of the study is that the Kinect can be used to assess breathing depth compared to a spirometer and is able to differentiate between quiet breathing patterns and breathing under different disturbances and loads.

There is a strong and highly significant correlation between the tidal volume recorded by the spirometer and the depth recorded by the Kinect. This relation between the two signals is not to be influenced by wearing a t‐shirt.

The effect of various disturbances on the respiratory patterns as captured by the Kinect is in line with the results found in the literature. For CLs, it has been highlighted that a mental cognitive task decreases the tidal volume,^24^ whereas an oral task will increase it.^25^ We observed the same changes in respiratory patterns with the Kinect for both types of disturbance. However, the decrease of the tidal volume was just above the significance level (*p* = 0.056) for the mental cognitive task (the dual‐task paradigm during part 3 of the validation).

Like with the CL, we were able to observe the same findings as Hostettler et al. for the IL.^26^ There is a significant increase in the depth (*p* < 0.001) with a 15 cm H_2_O IL.

Although the respiratory rate is increased with CL, we found a decrease in MV_like_, which is opposite to the findings of previous studies.^27^ In our study, the subjects seem to breathe more superficially when a CL is added, and this can result in a decrease of the minute ventilation. However, as expected, the IL increases the MV.^28^

We also assessed the thoracoabdominal motions during various tasks (part 3). The thoracoabdominal phase angle provides important information about the synchrony between these two compartments. In healthy subjects, there are differences in the contribution of the two compartments depending on sex (women have a more thoracic breathing) and age (reduction of the thoracic contribution and increase of the abdominal contribution in older).^29^ It is especially important in clinics to visualise asynchrony,^30^ or the effect of changing positions,^7^ during rehabilitation.^8^ The thoracoabdominal asynchrony is studied in many diseases such as COPD^7^, stroke^31^ or amyotrophic lateral sclerosis.^32^ This paradoxical breathing is, for example, linked to hyperinflation and exertional dyspnoea in COPD. These examples highlight the interest of this type of measurement for the evaluation of dyspnoea or respiratory insufficiency. In healthy subjects, females engage in more thoracic breathing than males.^25^ We observed similar patterns in our study in part 3. The trend angle between the thorax and abdomen for females is superior at 45°, thereby indicating thoracic breathing. For males, the result is the opposite. During the oral task, the thorax motion was more important than the abdomen. A previous study also showed the predominance of rib cage displacement during speech.^33^ Our rationale is that even if the subject does not say a word during a mental task, he/she starts a speech movement. During the intimation of speech, the thoracic volume increases while the abdominal volume decreases relative to relaxation.^34^ The thoracoabdominal phase angle provides important information about the synchrony between these two compartments. In healthy subjects, there are differences in the contribution of the two compartments depending on sex and age.^29^ In patients, studying thoracoabdominal angles allows, among other things, to demonstrate paradoxical breathing.

The last aspect that we evaluated was the instrumental effect. The instrumental effect describes the modification of breathing patterns when being observed and breathing throughout mouthpiece. It has been shown that breathing through a mouthpiece with a nose clip can increase the tidal volume by 29%.^35^ The new marker‐free techniques for respiratory patterns limited this effect.^1^ However, with a 10 cm Visual Analogue Scale, we observed a high score (5/10) for awareness of breathing. This perception may induce a modification of breathing patterns. Furthermore, when we induced a distraction with a cognitive task, we observed a decrease in the awareness of breathing (2/10, *p* < 0.002). This seems to indicate an important Hawthorne effect that cannot be neglected when performing breathing assessments and pattern evaluations. However, one of the added values of this system is that it is fully automated, and an evaluation can be carried out automatically without the presence of a health‐care professional. This is important not only to minimise the above‐mentioned Hawthorne effect but also in the general context of clinicians facing increasing financial and time constraints and, therefore, less time for face‐to‐face consultations with patients. Consequently, the present type of assessment that might be of particular benefit is the context of COVID‐19 crisis.

One of the limitations of this study is that the system required a Kinect sensor but other 3D cameras (e.g. Orbbec Astra Pro™ and Asus Xtion sensors™) or other affordable devices (e.g. multiple RGBD cameras) could be used instead of the Kinect, requiring only minor modifications to the software.

In this study, we validated the use of the Kinect to perform breathing assessments on patients suffering from chronic respiratory diseases. We also showed that the system can be used to evaluate modifications of breathing patterns induced by various loads and disturbances in healthy subjects mimicking the situations encountered by patients during their crisis phases. The proposed method is affordable, easy to use, and fully automated and could, therefore, be used to monitor the evolution of patients during the rehabilitation process or to perform a longitudinal follow‐up and monitor the efficacy of the prevention programme.

## AUTHOR CONTRIBUTIONS

OVH conceived the idea, performed data collection and data verification, drafted the manuscript and compiled edits. BB contributed to the design of the analysis, statistical analysis and revisions of the manuscript, and compiled edits. VA, AVM, DL, AL and VF contributed to revisions of the manuscript. AD, OD and RE conceived the idea, the python code and the analysis software.

## CONFLICT OF INTEREST STATEMENT

The authors do not have any conflict of interest to disclose.

## ETHICS STATEMENT

This study was approved by the Ethical Committee of Erasme Hospital (B406201734629, B406201733566, and B406201838283 for parts 1–3, respectively), and written informed consent was obtained from all subjects prior to their participation.

## Data Availability

The data that support the findings of this study are available from the corresponding author, OVH, upon reasonable request.

## References

[1] Niérat MC , Dubé BP , Llontop C , et al. Measuring Ventilatory activity with structured light plethysmography (SLP) reduces instrumental observer effect and preserves tidal breathing variability in healthy and COPD. Front Physiol. 2017;8:316. doi:10.3389/fphys.2017.00316 28572773 PMC5435806

[2] Fiamma MN , Samara Z , Baconnier P , Similowski T , Straus C . Respiratory inductive plethysmography to assess respiratory variability and complexity in humans. Respir Physiol Neurobiol. 2007;156(2):234‐239. doi:10.1016/j.resp.2006.12.001 17251070

[3] Raoufy MR , Ghafari T , Darooei R , et al. Classification of asthma based on nonlinear analysis of breathing pattern. PLoS ONE. 2016;11(1):e0147976. doi:10.1371/journal.pone.0147976 26824900 PMC4732950

[4] Aliverti A , Stevenson N , Dellacà RL , Lo Mauro A , Pedotti A , Calverley PMA . Regional chest wall volumes during exercise in chronic obstructive pulmonary disease. Thorax. 2004;59(3):210‐216. doi:10.1136/thorax.2003.011494 14985554 PMC1746979

[5] Sharp C , Soleimani V , Hannuna S , et al. Toward respiratory assessment using depth measurements from a time‐of‐flight sensor. Front Physiol. 2017;8:65. doi:10.3389/fphys.2017.00065 28223945 PMC5293747

[6] Hammer J , Newth CJL . Assessment of thoraco‐abdominal asynchrony. Paediatr Respir Rev. 2009;10(2):75‐80. doi:10.1016/j.prrv.2009.02.004 19410206

[7] Priori R , Aliverti A , Albuquerque AL , Quaranta M , Albert P , Calverley PMA . The effect of posture on asynchronous chest wall movement in COPD. J Appl Physiol. 2013;114(8):1066‐1075. doi:10.1152/japplphysiol.00414.2012 23412901

[8] Bernardi E , Pomidori L , Bassal F , Contoli M , Cogo A . Respiratory muscle training with normocapnic hyperpnea improves ventilatory pattern and thoracoabdominal coordination, and reduces oxygen desaturation during endurance exercise testing in COPD patients. Int J Chron Obstruct Pulmon Dis. 2015;10:1899‐1906. doi:10.2147/COPD.S88609 26392764 PMC4573075

[9] Perez A , Mulot R , Vardon G , Barois A , Gallego J . Thoracoabdominal pattern of breathing in neuromuscular disorders. Chest. 1996;110(2):454‐461. doi:10.1378/chest.110.2.454 8697851

[10] Clark RA , Mentiplay BF , Hough E , Pua YH . Three‐dimensional cameras and skeleton pose tracking for physical function assessment: a review of uses, validity, current developments and Kinect alternatives. Gait Posture. 2019;68:193‐200. doi:10.1016/j.gaitpost.2018.11.029 30500731

[11] Silverstein E , Snyder M . Comparative analysis of respiratory motion tracking using Microsoft Kinect v2 sensor. J Appl Clin Med Phys. 2018;19(3):193‐204. doi:10.1002/acm2.12318 PMC5978561 29577603

[12] Bae M , Lee S , Kim N . Development of a robust and cost‐effective 3D respiratory motion monitoring system using the Kinect device: accuracy comparison with the conventional stereovision navigation system. Comput Methods Programs Biomed. 2018;160:25‐32. doi:10.1016/j.cmpb.2018.03.027 29728243

[13] Ostadabbas S , Sebkhi N , Zhang M , et al. A vision‐based respiration monitoring system for passive airway resistance estimation. IEEE Trans Biomed Eng. 2016;63(9):1904‐1913. doi:10.1109/TBME.2015.2505732 26660514

[14] Windows Development Center . Kinect Hardware . Accessed July 1, 2019. https://developer.microsoft.com/en-us/windows/kinect/hardware

[15] Assessment and calibration of a RGB‐D camera (Kinect v2 Sensor) towards a potential use for close‐range 3D modeling. 10.3390/rs71013070

[16] Penne J , Schaller C , Hornegger J , Kuwert T . Robust real‐time 3D respiratory motion detection using time‐of‐flight cameras. Int J CARS. 2008;3(5):427‐431. doi:10.1007/s11548-008-0245-2

[17] Bonnechère B , Jansen B , Salvia P , et al. Determination of the precision and accuracy of morphological measurements using the Kinect™ sensor: comparison with standard stereophotogrammetry. Ergonomics. 2014;57(4):622‐631. doi:10.1080/00140139.2014.884246 24646374

[18] Soleimani V , Mirmehdi M , Damen D , et al. Remote pulmonary function testing using a depth sensor. In: *2015 IEEE Biomedical Circuits and Systems Conference (BioCAS)*. 2015;1‐4. 10.1109/BioCAS.2015.7348445

[19] Halpin DMG , Decramer M , Celli BR , Mueller A , Metzdorf N , Tashkin DP . Effect of a single exacerbation on decline in lung function in COPD. Respir Med. 2017;128:85‐91. doi:10.1016/j.rmed.2017.04.013 28610675

[20] Bonnechère B , Van Vooren M , Bier JC , et al. The use of mobile games to assess cognitive function of elderly with and without cognitive impairment. J Alzheimers Dis. 2018;64(4):1285‐1293. doi:10.3233/JAD-180224 29991133

[21] Garfinkel SN , Seth AK , Barrett AB , Suzuki K , Critchley HD . Knowing your own heart: distinguishing interoceptive accuracy from interoceptive awareness. Biol Psychol. 2015;104:65‐74. doi:10.1016/j.biopsycho.2014.11.004 25451381

[22] Nierat MC , Demiri S , Dupuis‐Lozeron E , et al. When breathing interferes with cognition: experimental inspiratory loading alters timed up‐and‐go test in normal humans. PLoS ONE. 2016;11(3):e0151625. doi:10.1371/journal.pone.0151625 26978782 PMC4792478

[23] Van Hove O , Pichon R , Pallanca P , et al. Influence of speech and cognitive load on balance and timed up and go. Brain Sci. 2022;12(8):1018. doi:10.3390/brainsci12081018 36009081 PMC9405849

[24] Hagio K , Obata H , Nakazawa K . Effects of breathing movement on the reduction of postural sway during postural‐cognitive dual tasking. PLoS ONE. 2018;13(5):e0197385. doi:10.1371/journal.pone.0197385 29813100 PMC5973601

[25] Binazzi B , Lanini B , Bianchi R , et al. Breathing pattern and kinematics in normal subjects during speech, singing and loud whispering. Acta Physiol (Oxf). 2006;186(3):233‐246. doi:10.1111/j.1748-1716.2006.01529.x 16497202

[26] Hostettler S , Illi SK , Mohler E , Aliverti A , Spengler CM . Chest wall volume changes during inspiratory loaded breathing. Respir Physiol Neurobiol. 2011;175(1):130‐139. doi:10.1016/j.resp.2010.10.001 20937414

[27] Grassmann M , Vlemincx E , von Leupoldt A , Mittelstädt JM , Van den Bergh O . Respiratory changes in response to cognitive load: a systematic review. Neural Plast. 2016;2016:8146809. doi:10.1155/2016/8146809 27403347 PMC4923594

[28] da Fonsêca JDM , Resqueti VR , Benício K , Fregonezi G , Aliverti A . Acute effects of inspiratory loads and interfaces on breathing pattern and activity of respiratory muscles in healthy subjects. Front Physiol. 2019;10. Accessed October 17, 2022. https://www.frontiersin.org/articles/10.3389/fphys.2019.00993 10.3389/fphys.2019.00993 PMC6688654 31427989

[29] Mendes LPDS , Vieira DSR , Gabriel LS , et al. Influence of posture, sex, and age on breathing pattern and chest wall motion in healthy subjects. Braz J Phys Ther. 2020;24(3):240‐248. doi:10.1016/j.bjpt.2019.02.007 PMC7253877 30967355

[30] Pereira PAB , Aho VTE , Paulin L , Pekkonen E , Auvinen P , Scheperjans F . Oral and nasal microbiota in Parkinson's disease. Parkinsonism Relat Disord. 2017;38:61‐67. doi:10.1016/j.parkreldis.2017.02.026 28259623

[31] Lima ÍN , Fregonezi GA , Melo R , et al. Acute effects of volume‐oriented incentive spirometry on chest wall volumes in patients after a stroke. Respir Care. 2014;59(7):1101‐1107. doi:10.4187/respcare.02651 24222704

[32] Sarmento A , Fregonezi G , Dourado‐Junior MET , et al. Thoracoabdominal asynchrony and paradoxical motion in middle stage amyotrophic lateral sclerosis. Respir Physiol Neurobiol. 2019;259:16‐25. doi:10.1016/j.resp.2018.06.012 29969705

[33] Estenne M , Zocchi L , Ward M , Macklem PT . Chest wall motion and expiratory muscle use during phonation in normal humans. J Appl Physiol. 1990;68(5):2075‐2082. doi:10.1152/jappl.1990.68.5.2075 2361909

[34] Stathopoulos ET , Hoit JD , Hixon TJ , Watson PJ , Solomon NP . Respiratory and laryngeal function during whispering. J Speech Hear Res. 1991;34(4):761‐767. doi:10.1044/jshr.3404.761 1956183

[35] Gilbert R , Auchincloss JH , Brodsky J , Boden W . Changes in tidal volume, frequency, and ventilation induced by their measurement. J Appl Physiol. 1972;33(2):252‐254. doi:10.1152/jappl.1972.33.2.252 5054434

